# Centering Ability and Influence of Experience When Using WaveOne Single-File Technique in Simulated Canals

**DOI:** 10.1155/2012/206321

**Published:** 2012-10-16

**Authors:** Mathieu Goldberg, Sandrine Dahan, Pierre Machtou

**Affiliations:** Université paris 7 Diderot, UFR d'odontologie, 5 Rue Garancière, 75006 Paris, France

## Abstract

*Introduction*. WaveOne is a single endodontic instrument that reciprocates with a dedicated motor to shape root canal systems. The present study assessed the centering ability of this simplified protocol along with the effect of experience in simulated plastic canals. *Methods*. One experienced operator shaped two groups of simulated canals. Groups 1 and 3 each comprised 30 blinded L-shaped canals or S-shaped canals, respectively. Photographs were taken before and after shaping and digitally assessed centering after superimposition of the pictures. Time of shaping, number of passes, canal aberrations, and instrument degradation were recorded. In group 2 shaping was done on 30 blinded L-shaped canals by 30 different students with no prior experience with WaveOne. *Results*. All three groups yielded satisfactory, reproducible shaping. Centering ratios were low and homogeneous in all groups, with no significant differences between the experienced operator and the students. Apical transportation values were very low (≤0.138 mm) with no instances of blockage or separation. The average shaping time was short (43 to 101,6 sec). *Conclusions*. Within the limits of the study, the WaveOne instrument had excellent centering ability with a low risk of fracture or blockage and a short shaping time, regardless of the operator's level of experience.

## 1. Introduction

Root canal shaping is a key stage of endodontic treatment; when performed properly, it is a predictive factor for success. Ideally, root canal shaping should create a continuous tapered preparation from crown to apex while maintaining the original path of the canal and keeping the foramen size as small as practical [1]. These objectives can be difficult to achieve by using stainless steel hand instrumentation [2]. Thus, the introduction of rotary nickel titanium (NiTi) instrumentation was an important step in optimal root canal shaping [3]. This approach is faster, safer, and more reproducible, with a lower risk of procedural errors compared to hand instrumentation [4]. However, the risk of fracture of NiTi rotary files is still a concern amongst clinicians [5, 6].

 A 2008 publication described a single file-technique using asymmetric reciprocation [7]. The objectives of this new technique were to reduce the working time and cost and improve safety of the shaping procedure. Recently, the WaveOne (Dentsply-Maillefer, Ballaigues, Switzerland), a reciprocating file system with a dedicated motor was launched on the market. To the best of our knowledge, few scientific data are currently available regarding this new shaping system [8–12]. Centering ability is influenced both by the design of the instrument (taper, flexibility, and type of alloy) and the root canal anatomy. The straighter the root canal is, the less the instrument receives constraint and the more centered it is. Bürklein et al. [10] have shown that root canal shaping with WaveOne instrument can be performed with a good centering ability in regularly curved canals of extracted teeth. Recently, Kim et al. [11, 12], using microcomputed tomography, did not find any difference in transportation values between Protaper F2 and WaveOne and confirmed the good behaviour of WaveOne. 

The aim of the study was to assess the centering ability of WaveOne in simulated plastic L-curved root canals and to compare the effect of experience on results obtained by an experienced operator with those of novice students. In addition, some parameters such as taper, aberrations, preparation time, and quantity of removed resin will be analyzed. Furthermore a third group of S-shaped canals was prepared by the same experienced operator in order to assess the centering ability of WaveOne in a more complex situation.

## 2. Materials and Methods

### 2.1. Experimental Design

**Figure 1:**
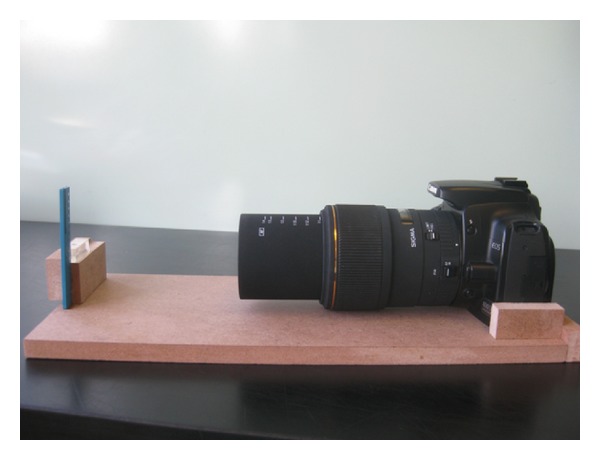
Set-up.

Three groups each prepared 30 simulated canals with a WaveOne 25.08 primary file. The centering ability, apical transportation, maintenance of apical diameter, and taper were assessed within each group. Group 1 comprised 30 curved L-shaped simulated canals in plastic blocks (Endo Training Bloc-L; Dentsply-Maillefer, Ballaigues, Switzerland), which were prepared by an operator who had previously mastered this technique (MG). All of the simulated canals were standardized as follows: they were 16.5 mm long, the foramen diameter was 0.15 mm and the initial taper was .02. The radius and angle of curvature were measured using Pruett's technique [13] and were 4.5 mm and 60°, respectively. Group 2 comprised similar L-shaped simulated canals, but the procedure was performed by 30 fifth-year students who had no previous experience with this technique. The students received a brief training, which included written instructions, watching a short video clip, and training on a single clear simulated canal. Group 3 comprised 30 S-shaped simulated plastic canals (Endo Training Bloc-S; Dentsply-Maillefer—Ballaigues—Switzerland), which were prepared by the same experienced operator (MG). The S-shaped canals were 16.5 mm long, the foramen diameter was 0.15 mm, and the initial taper was .02. The radius and angle of curvature, respectively, were 5 mm and 35° for the first curvature and 4.5 mm and 30° for the second curvature. All of the plastic blocks were covered with aluminum foil during the preparation. A specific platform was built to take pictures of the canals before and after shaping (Figure 1). This set-up allowed precise camera (CANON 400D with SIGMA 105 mm F2.8 DG Macro EX lens) and plastic block repositioning. For calibration, an endo ruler (Dentsply-Maillefer, Ballaigues, Switzerland) was fixed adjacent to the plastic block and holes were used as a size reference with 600% magnification. A light source was placed behind the plastic blocks to create back lighting. Before shaping, canals were irrigated with iodine (Betadine Buccale; Meda Pharma, France) to make them clearly visible on the preoperative pictures.

**Figure 2:**
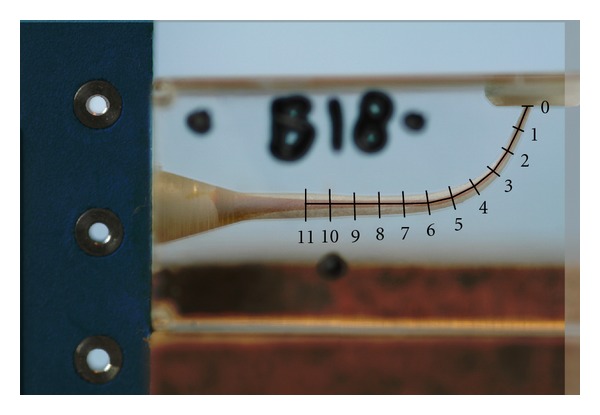
L-shaped simulated canals: pictures superimposed.

**Figure 3:**
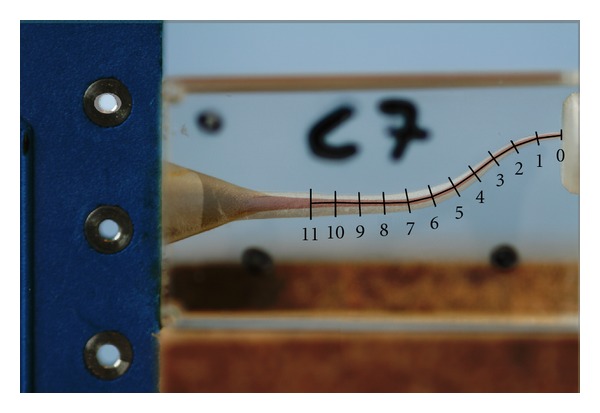
S-shaped simulated canals: pictures superimposed.

The preoperative and postoperative pictures were surimposed to analyze the shaping ability of the WaveOne instrument (Figures 2 and 3).

The shaping technique followed the manufacturer's instructions with a slight modification in order to improve safety and predictability. A light pecking action was used to engage the file tip followed by a 2.5–3 mm passive penetration cycle. After each cycle the canal was irrigated with 1 mL of isopropyl alcohol and flutes of the instrument were cleaned with a moistened gauze. According to the directions for use, cleaning the flutes is mandatory in order to maintain cutting efficiency and to avoid exerting more apical pressure on the instrument potentially leading to much engagement of the file and a higher risk of fracture. The 2.5–3 mm cycles were repeated until working length is reached. 

The canals were first scouted with a #10 K-file (FlexoFile; Dentsply-Maillefer, Ballaigues, Switzerland) to check patency and precisely determine the working length. Before shaping, a drop of EDTA gel (Glyde File Prep; Dentsply-Maillefer, Ballaigues-Switzerland) was placed inside the coronal reservoir for lubrication. The preparation was performed with a single rotary file, the WaveOne Primary (lot 7134330; Dentsply-Maillefer, Ballaigues, Switzerland), and the preprogrammed motor (X-Smart Plus-Dentsply-Maillefer, Ballaigues, Switzerland), using a specific movement of reciprocation: 170° CCW and 50° CW at a speed equivalent to 350 rpm [11, 12] according to the aforementioned protocol. The number of passes and the total working time was recorded for each block. Time of shaping included instrumentation, irrigation, and instrument cleaning.

Images were analysed with Photoshop CS5 (ADOBE), and Image J 1.43u software (National Institutes of Health, http://rsb.info.nih.gov/ij/download.html) was used to make measurements every one millimeter from D0 to D11. Three measurements were recorded with 0.001 mm precision and 600% magnification at each level for a total of 36 measurements per canal. We measured (1) the distance between the upper limit of the initial canal and the upper limit of the instrumented canal (*X*_sup⁡_), (2) the distance between the inferior limit of the initial canal and the inferior limit of the instrumented canal (*X*_inf⁡_), and (3) the width of the shaped canal (*Y*). 

Using the following equations [14], these data provided a quantitative and qualitative evaluation of the shaping ability of WaveOne: (1) total amount of resin removal, *X*_sup⁡_ + *X*_inf⁡_; (2) amount and direction of transportation, *X*_sup⁡_ − *X*_inf⁡_; (3) centering ratio (*X*_sup⁡_ − *X*_inf⁡_)/*Y* [15]. We also recorded aberrations (e.g., occurrence of blockage, apical zip, and ledges) and working time.

### 2.2. Statistical Analysis

Statistical analysis was performed with SigmaStat v2.03 software (Jandel Corporation). Data were compared using Student's *t*-test (*α* = 0.05). The normal distribution of variables was controlled with the Kolmogorov-Smirnov test, and the nonparametric Mann-Whitney test was used for data that were not normally distributed. The Kruskal-Wallis test was used to compare data regarding the number of instrument passes and the time of shaping.

## 3. Results

Neither blockage nor instrument separation occurred. Therefore, all of the canals were used for evaluation and statistical analyses

### 3.1. Measurement of Material Removed

A steady and increasing amount of plastic was removed in the same proportions among the three groups (Table 1). Therefore, a continuous tapered preparation was achieved regardless of operator experience (group 1 & 2) and canal anatomy (group 3).

Apical mean diameters at D0 after shaping were 0.297, 0.307, and 0.288 mm, respectively, for groups 1, 2, and 3. We did not find any statistically significant differences in the apical mean diameters among the three. The foramen diameters were slightly larger than the instrument tip size (0.25 mm). The transportation of the foramen being almost nonexistent, the increase in foramen size should likely be associated with a slight overinstrumentation. Since the WaveOne instrument has a .08 apical taper on the last three millimeters, overinstrumentation can be calculated according to the following formula:

(1) $$\begin{array}{c} \text{OI}=\frac{\left(\text{FS}-0.25\right)}{0.8}, \end{array}$$

with OI for over instrumentation and FS for foramen size observed. 

Therefore, overinstrumentation was 0.59, 0.72, and 0.48 mm, respectively, in groups 1, 2, and 3.

### 3.2. WaveOne Centering Ability

**Figure 4:**
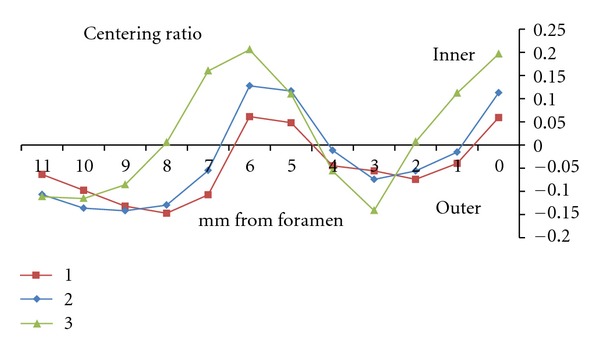
Total amount means of removed resin (mm) at the different levels after root canal preparation.

**Figure 5:**
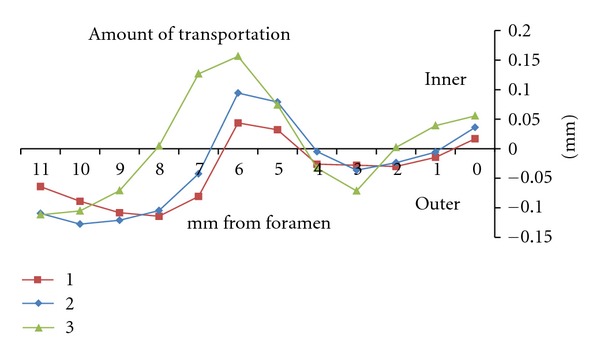
Centering ratio means at the different levels after root canal preparation.

Macroscopic observation of the superimposed pre- and postoperative photographs in all three groups revealed that the centering ability of the WaveOne was excellent (Figures 2 and 3). The closer the centering ratio was to zero, the more centered the shaping was. The mean values of the ratio (Table 2) were close to zero and relatively homogeneous in all three groups. When comparing the ratio at each point in groups 1 and 2, no statistically significant difference was found, except at points 5 and 7 (*P* < 0.001), which corresponded to curve initiation (Figures 4 and 5).

At the foramen level, there were no statistically significant differences between groups 1 and 2, but we did not note any differences between groups 1 and 3 (*P* < 0.001) and 2 and 3 (*P* < 0.01). Nevertheless, in group 3, the centering ratio remained very close to zero.

### 3.3. Preparation Time

All of the results are presented in Table 3.

### 3.4. Instrument Aberration

Macroscopic observation of the instruments after shaping revealed that of the 90 instruments used, eight were deformed. All of the damaged instruments belonged to group 3. Untwisting of the flutes was always located in the apical one-third of the instrument.

## 4. Discussion

A new instrument has to fulfill the well-agreed shaping objectives proposed by Schilder in [1]. Therefore, the aim of the study was to evaluate WaveOne shaping ability when used by an experienced operator and novice students in blinded L-shaped canal. Blinded S-shaped canals were then prepared by the experienced operator to assess the behaviour of this new endodontic instrument in a more complex anatomy. In both situations, the manufacturer mode of use was slightly modified. Indeed, according to our clinical experience, care should be taken not to use a pumping action during the shaping procedure. A pumping action with a reciprocating motion has a tendency to pack debris beyond the tip and favors blockage and pressure leading to aberrations. Besides, extrusion of debris is a frequent occurrence in this approach [16].

The present study demonstrated an excellent shaping and centering ability of WaveOne single-file instrumentation in simulated plastic canals regardless of operator experience and canal anatomy. To our knowledge, only two studies have assessed the shaping ability of the WaveOne primary instrument with a dedicated preprogrammed reciprocating motor [10–12].

Previous studies have investigated single file reciprocation using the Protaper F2 with a ATR Technika motor [17, 18]. The use of simulated canals in resin blocks has been widely described in the literature [14, 15] and this experimental model has been shown to be scientifically valid [15]; it allows for good standardization and avoids variables that may be caused by differences in the anatomy: shape, size, taper, degree, location and radius of curvature, and dentin hardness of extracted teeth [14, 17]. However, one must be cautious about extrapolating the results to natural teeth [14]. As opposed to natural teeth, plastic canals are always patent and are manufactured with an initial taper equivalent to an ISO standard size 15 throughout their length. According to the manufacturer mode of use, the creation a secured glide path with a loose *≠* 10 K file. However, a recent study has shown that the WaveOne primary file, if used after a previous glide path made with PathFiles, produced less modification in canal curvature suggesting hence that the presence of a bigger canal hole improves the performance of the instrument [9]. Nevertheless, the PathFiles were not used in the present study. Superimposition of canal outlines using digital photography is an accurate method to assess reproducibility and measurement of transportation [14, 19]. This model is widely used in the literature, but has some limitations [19]. Currently, microcomputed tomography is a model closer to clinical reality but it does not allow a direct and objective comparison between operators. This technique has been used by Loizides et al. [20] to compare canal shaping results between Hero-shaper and Protaper instrument. In a recent study, Kim et al. ([11, 12], in press) using micro-CT did not find any difference in transportation between WaveOne and Protaper F2. Few studies have used the centering ratio (*X*_sup⁡_ − *X*_inf⁡_)/*Y* [14, 15], which is more accurate than measuring the amount of removed material. Indeed, most previous studies did not include the final diameter *Y* in the calculation, and instead considered only the amount of transportation. Although a direct comparison was not made, the apical transportation values obtained in the present study appeared to be much lower than those of other studies using rotary instrumentation in plastic canals, [2, 14, 15, 21] (Table 1 and Figure 5).

The centering ratios were low and similar in all three groups. The lowest ratios in groups 1 and 2 (L-shaped canals) were found at points 1 and 4 and corresponded to the straight portion of the canal after curvature. The highest ratios were found at points 8–10 and corresponded to the straight portion before the curvature. The lack of consistency found among operators at points 8–10 is most likely due to the inconstant position of the access zone, since some discrepancies exist on plastic blocks between the access zone and canal axis. Canal transportation was similar in both groups: external before curve initiation and internal at curve initiation and at the apical level. A very slight transportation occurred toward the internal wall at the foramen level, indicating that the reciprocating motion and M-wire alloy provided excellent centering action, despite the use of a 0.8-tapered instrument. 

In group 3 (S-shaped canals), the lowest centering ratios were found at points 2, 4, and 8 and corresponded to the straight portion of the canal. The highest ratios were found at points 0, 3, 6, and 7 and corresponded to the foramen and initiating zones of the curvatures. Canal transportation was external before both curve initiations and internal at the first curve initiation, at the end of the second curve and at the foramen level.

In contrast with other similar studies, measurements were done at the foramen level instead of 1 mm from the foramen, and they revealed a nonsignificant amount of transportation. Interestingly, the apical diameters exceeded the tip size of the WaveOne Primary instrument: 0.25 mm. This may be due to slight overinstrumentation caused by a small decrease in the working length that can be offset by a brushing action during the shaping procedure of the coronal 2/3 and then a new determination of the working length [8]. It could also be due to the difficulty of maintaining a precise working length with mechanized instrumentation. 

Nickel-titanium (Ni-Ti) rotary files are an important adjunct in clinical endodontics [3]. They produce smooth-tapered canal preparations with reproducible results, even when performed by novice students [22]. Compared to manual stainless steel instrumentation, inexperienced operators achieved better canal preparations with rotary Ni-Ti instruments but rotary preparation was associated with significantly more fractures [23]. Most studies comparing experienced and novice operators found differences in shaping ability and efficiency [22, 24]. Therefore, practice and experience are indispensable for safe application of this technique [25]. However, no significant differences were found in the present study, suggesting that there is a very short learning curve for the reciprocation technique and that it is suitable for teaching purposes, especially considering that all of the shaping was done blindly.

The shaping time, including irrigation and cleaning of the flutes, was slightly longer for the students: 101 sec on average compared to 43 sec for the experienced operator (Table 2). Nevertheless, the shaping time even for the students seem to be much shorter than results obtained in similar studies using rotary instruments in simulated canals (e.g., Race, Hero 642, and Pro Taper), which were 2–10 min [2, 21]. This is consistent with the results of previous studies that assessed single-file reciprocation with the Protaper F2 [7, 17, 18, 26], You et al. [27] and corroborates those of Bürklein et al. [10], who compared WaveOne and Reciproc versus Mtwo and Protaper rotary technique during the preparation of curved root canals in extracted teeth. For an adequate disinfection, antiseptic irrigation is critical and can be only effective at the completion of a good shaping procedure [28]. Obviously, sufficient time and activation of irrigants should be devoted to this critical step to compensate the reduced shaping time.

Unlike the Roane technique [19] single-file reciprocation creates a cutting action that is much greater than disengagement, thereby allowing better apical progression and higher efficiency. 

Aberrations and fractures may occur when rotary instrumentation is used. However, with reciprocation and within the conditions of this study, no fracture or blockage were observed, even for inexperienced students. Only a few cases of untwisting of the apical flutes could be noticed in S-curved canals during the last steps of the shaping procedure. This corroborates the results of Kim et al. [11, 12] who showed that WaveOne and Reciproc (Dentsply-VDW, Munich, Germany) demonstrated significantly higher cyclic fatigue and torsional resistance than ProTaper F2.

## 5. Conclusion

Within the limits of this study, it was found that WaveOne single-file reciprocation had a very good centering ability when shaping simulated canals regardless of the level of operator experience and canal anatomy. Besides, the overall shaping time was fast, and there was a reduced risk of aberrations, blockages, and fractures. This new technique appeared to be promising, but further clinical and laboratory investigations are needed to confirm the results obtained in this study.

## Figures and Tables

**Table 1:** Total quantity means of removed resin (mm) and Standard Deviations (SD) at the different measure points after root canal preparation.

	Measure points (in mm from the foramen)											
	*X*_sup⁡_ + *X*_inf⁡_											
	0	1	2	3	4	5	6	7	8	9	10	11
Group 1												
Mean	0,128^a^	0,177^a^	0,215^a^	0,289^a^	0,370^a^	0,426^a^	0,464^b^	0,488^b^	0,498^b^	0,519^b^	0,564^a^	0,575^a^
SD	0,042	0,035	0,037	0,043	0,042	0,028	0,017	0,020	0,026	0,042	0,051	0,045
Group 2												
Mean	0,138^a^	0,193^a^	0,229^a^	0,303^a^	0,369^a^	0,431^a^	0,484^b^	0,508^b^	0,525^b^	0,540^b^	0,568^a^	0,564^a^
SD	0,047	0,036	0,041	0,048	0,043	0,045	0,034	0,030	0,029	0,042	0,054	0,044
Group 3												
Mean	0,137^a^	0,179^c^	0,215^c^	0,302^c^	0,367^c^	0,454^c^	0,524^c^	0,543^c^	0,537^c^	0,542^c^	0,583^c^	0,600^c^
SD	0,037	0,019	0,027	0,030	0,030	0,030	0,027	0,032	0,025	0,029	0,040	0,027

^a^ *P* > 0.05: On exponent, the identical letters illustrate the values which do not present significant difference

^b^ *P* < 0.05: On exponent, the identical letters illustrate the values which present a significant difference

^c^—: Values not having been the object of statistical analysis of differences.

**Table 2:** Centering ratio means and Standard Deviation (SD) at the different levels in groups 1, 2, and 3.

	Measure points (in mm from the foramen)											
	*R*											
	0	1	2	3	4	5	6	7	8	9	10	11
Group 1												
Mean	0,059^a,c^	−0,040^a^	−0,074^a^	−0,056^a^	−0,045^a^	0,048^b^	0,061^b^	−0,107^b^	−0,147^a^	−0,132^a^	−0,098^a^	−0,064^a^
SD	0,087	0,077	0,077	0,062	0,054	0,059	0,050	0,041	0,042	0,051	0,065	0,088
Group 2												
Mean	0,113^a,b^	−0,015^a^	−0,056^a^	−0,074^a^	−0,011^a^	0,116^b^	0,128^b^	−0,055^b^	−0,130^a^	−0,142^a^	−0,136^a^	−0,107^a^
SD	0,135	0,076	0,070	0,069	0,078	0,088	0,071	0,055	0,050	0,058	0,085	0,105
Group 3												
Mean	0,196^b,c^	0,112^d^	0,007^d^	−0,141^d^	−0,056^d^	0,110^d^	0,206^d^	0,160^d^	0,005^d^	−0,086^d^	−0,115^d^	−0,112^d^
SD	0,107	0,069	0,075	0,066	0,061	0,056	0,045	0,056	0,056	0,044	0,042	0,044

^a^ *P* > 0.05: On exponent, the identical letters illustrate the values which do not present significant difference

^b,c^ *P* < 0.05: On exponent, the identical letters illustrate the values which present a significant difference

^d^—: Values not having been the object of statistical analysis of différences.

**Table 3:** Average shaping time (sec) and number of passes.

	Shaping time (sec)		Number of passes	
	Average	Standard deviation	Average	Standard deviation
Group 1	43	5.8	4.8	0.4
Group 2	101.6	27.7	5.9	1.4
Group 3	53.1	7.3	6.7	0.8

Differences were statistically significant among all three groups for shaping time and number of passes (*P* < 0.001).

## References

[1] Schilder H (1974). Cleaning and shaping the root canal. *Dental Clinics of North America*.

[2] Schäfer E (2001). Shaping ability of Hero 642 rotary nickel-titanium instruments and stainless steel hand K-Flexofiles in simulated curved root canals. *Oral Surgery, Oral Medicine, Oral Pathology, Oral Radiology, and Endodontics*.

[3] Bergmans L, Van Cleynenbreugel J, Wevers M, Lambrechts P (2001). Mechanical root canal preparation with NiTi rotary instruments: rationale, performance and safety. Status Report for the American Journal of Dentistry. *American Journal of Dentistry*.

[4] Guelzow A, Stamm O, Martus P, Kielbassa AM (2005). Comparative study of six rotary nickel-titanium systems and hand instrumentation for root canal preparation. *International Endodontic Journal*.

[5] Tzanetakis GN, Kontakiotis EG, Maurikou DV, Marzelou MP (2008). Prevalence and management of instrument fracture in the postgraduate endodontic program at the dental school of athens: a five-year retrospective clinical study. *Journal of Endodontics*.

[6] Parashos P, Messer HH (2006). Rotary NiTi instrument fracture and its consequences. *Journal of Endodontics*.

[7] Yared G (2008). Canal preparation using only one Ni-Ti rotary instrument: preliminary observations. *International Endodontic Journal*.

[8] Berutti E, Chiandussi G, Paolino DS (2011). Effect of canal length and curvature on working length alteration with WaveOne reciprocating files. *Journal of Endodontics*.

[9] Berutti E, Paolino DS, Chiandussi G (2012). Root canal anatomy preservation of WaveOne reciprocating files with or without glide path. *Journal of Endodontics*.

[10] Bürklein S, Hinschitza K, Dammaschke T, Schäfer E (2012). Shaping ability and cleaning effectiveness of two single-file systems in severely curved root canals of extracted teeth: reciproc and WaveOne versus Mtwo and ProTaper. *International Endodontic Journal*.

[11] Kim HC, Kwak SW, Shun-Pan Cheung G, Ko DH, Chung SM, Lee W (2012). Cyclic fatigue and torsional resistance of two new nickel-titanium instruments used in reciprocation motion: reciproc versus WaveOne. *Journal of Endodontics*.

[12] Kim HC, Hwang YJ, Jung DW, You SY, Kim HC, Lee W Micro-computed tomography and scanning electron microscopy comparisons of two nickel-titanium rotary root canal instruments used with reciprocating motion.

[13] Pruett JP, Clement DJ, Carnes DL (1997). Cyclic fatigue testing of nickel-titanium endodontic instruments. *Journal of Endodontics*.

[14] Ersev H, Yilmaz B, Ciftçioglu E, Ozkarsli SF (2010). A comparison of the shaping effects of 5 nickel-titanium rotary instruments in simulated S-shaped canals. *Oral Surgery, Oral Medicine, Oral Pathology, Oral Radiology and Endodontology*.

[15] Loizides A, Eliopoulos D, Kontakiotis E (2006). Root canal transportation with a Ni-Ti rotary file system and stainless steel hand files in simulated root canals. *Quintessence International*.

[16] Bürklein S, Schäfer E (2012). Apically extruded debris with reciprocating single-file and full-sequence rotary instrumentation systems. *Journal of Endodontics*.

[17] Varela-Patiño P, Ibañez-Párraga A, Rivas-Mundiña B, Cantatore G, Otero XL, Martin-Biedma B (2010). Alternating versus continuous rotation: a comparative study of the effect on instrument life. *Journal of Endodontics*.

[18] Paqué F, Zehnder M, De-Deus G (2011). Microtomography-based comparison of reciprocating single-File F2 ProTaper technique versus rotary full sequence. *Journal of Endodontics*.

[19] Madureira RG, Forner Navarro L, Llena MC, Costa M (2010). Shaping ability of nickel-titanium rotary instruments in simulated S-shaped root canals. *Oral Surgery, Oral Medicine, Oral Pathology, Oral Radiology and Endodontology*.

[20] Loizides AL, Kakavetsos VD, Tzanetakis GN, Kontakiotis EG, Eliades G (2007). A comparative study of the effects of two nickel-titanium preparation techniques on root canal geometry assessed by microcomputed tomography. *Journal of Endodontics*.

[21] Yang GB, Zhou XD, Zhang H, Wu HK (2006). Shaping ability of progressive versus constant taper instruments in simulated root canals. *International Endodontic Journal*.

[22] Baumann MA, Roth A (1999). Effect of experience on quality of canal preparation with rotary nickel-titanium files. *Oral Surgery, Oral Medicine, Oral Pathology, Oral Radiology, and Endodontics*.

[23] Sonntag D, Delschen S, Stachniss V (2003). Root-canal shaping with manual and rotary Ni-Ti files performed by students. *International Endodontic Journal*.

[24] Mesgouez C, Rilliard F, Matossian L, Nassiri K, Mandel E (2003). Influence of operator experience on canal preparation time when using the rotary Ni-Ti ProFile system in simulated curved canals. *International Endodontic Journal*.

[25] Mandel E, Adib-Yazdi M, Benhamou LM, Lachkar T, Mesgouez C, Sobel M (1999). Rotary Ni-Ti profile systems for preparing curved canals in resin blocks: influence of operator on instrument breakage. *International Endodontic Journal*.

[26] De-Deus G, Moreira EJL, Lopes HP, Elias CN (2010). Extended cyclic fatigue life of F2 ProTaper instruments used in reciprocating movement. *International Endodontic Journal*.

[27] You SY, Bae KS, Baek SH, Kum KY, Shon WJ, Lee W (2010). Lifespan of one nickel-titanium rotary file with reciprocating motion in curved root canals. *Journal of Endododontics*.

[28] Bronnec F, Bouillaguet S, Machtou P (2010). Ex vivo assessment of irrigant penetration and renewal during the final irrigation regimen. *International Endodontic Journal*.

